# Community-based screening enhances hepatitis B virus linkage to care among West African migrants in Spain

**DOI:** 10.1038/s43856-023-00420-8

**Published:** 2023-12-14

**Authors:** Camila A. Picchio, Daniel K. Nomah, Ariadna Rando-Segura, Maria Buti, Sabela Lens, Xavier Forns, Sergio Rodriguez Tajes, Emma Fernández, Javier Pamplona Portero, Carmen López Nuñez, Lena van Selm, Marina MacKinnon, Silvia G. Araujo, Elisa Martró, Francisco Rodríguez-Frías, Jeffrey V. Lazarus

**Affiliations:** 1 Barcelona Institute for Global Health (ISGlobal), Hospital Clínic, University of Barcelona, Barcelona, Spain; 2 Center for Epidemiological Studies on Sexually Transmitted Infections and HIV/AIDS in Catalonia (CEEISCAT), Department of Health, Generalitat of Catalonia, Badalona, Spain; 3 Microbiology Department, Vall d’Hebron Hospital Universitari, Barcelona, Spain; 4 CIBER Hepatic and Digestive Diseases (CIBERehd), Instituto Carlos III, Madrid, Spain; 5 Hospital Campus, Liver Unit, Hospital Universitari Vall d’Hebron, Barcelona, Spain; 6 Liver Unit, Hospital Clínic, IDIBAPS, University of Barcelona, Barcelona, Spain; 7 Department of Digestive Diseases, Hospital de Santa Caterina, Salt (Girona), Spain; 8 Department of Digestive Diseases, Hospital Trueta, Girona, Spain; 9 Microbiology Department, Laboratori Clínic Metropolitana Nord (LCMN), Hospital Universitario Germans Trias i Pujol, Institut d’Investigació Germans Trias i Pujol (IGTP), Badalona, Spain; 10 CIBER in Epidemiology and Public Health (CIBERESP), Instituto Carlos III, Madrid, Spain; 11 Liver Pathology Unit, Biochemistry and Microbiology Service, Hospital Universitari Vall d’Hebron, Barcelona, Spain; 12 CUNY Graduate School of Public Health and Health Policy (CUNY SPH), New York, NY USA

**Keywords:** Epidemiology, Hepatitis B

## Abstract

**Background:**

Chronic infection with HBV is responsible for >50% of all hepatocellular cancer cases globally and disproportionately affects sub-Saharan African (sSA) countries. Migration from these countries to Europe has increased substantially in recent years, posing unique challenges to health systems. The aim of this study was to carry out a community-based intervention to increase HBV screening, vaccination, and linkage to care among sSA migrants in Catalonia, Spain.

**Methods:**

This was a prospective cohort study. Participants ≥18 years were offered community-based HBV screening between 20/11/20 and 21/01/22. Rapid HBV testing and blood sample collection utilizing plasma separation cards were carried out and linkage to care was offered to all participants. HBV vaccination and post-test counseling were performed at a second visit in the community. The main outcome was the odds of those with current HBV infection being successfully linked to hepatology. Rates of completing the care cascade of this model were analyzed.

**Results:**

In the present study, 444 people undergo screening, with 50.6% of participants showing evidence of past or current HBV infection, including an HBsAg prevalence of 9.2%. Migrants with current HBV infection exhibit 5.2 times higher odds of successful linkage to care compared to those in need of post-test counseling or vaccination. The study achieves a successful linkage to care rate of 72% for all participants, with specialist appointments arranged within 15.5 days.

**Conclusions:**

This community-based HBV screening program provides evidence of a successful model for identifying and providing care, including vaccination, to west African migrants at high risk of HBV infection who may otherwise not engage in care.

## Plain language summary

A large proportion of hepatitis B virus (HBV) infections occur within countries in sub-Saharan Africa. With recent increased migration from these countries to Catalonia Spain, the prevalence of HBV is greater in migrants than in host populations. However, migrants face additional barriers when trying to access care. We developed a community-based care pathway to provide migrants in Catalonia with access to HBV testing, post-test counseling, vaccinations, and appointments with specialists when needed. The results showed that this strategy was successful in increasing testing, linkage to care, and vaccination among at-risk migrant populations in Catalonia, Spain. It may be worthwhile implementing this strategy on a wider scale and with other at-risk populations to reduce HBV infections and improve outcomes.

## Abstract (web summary)

Picchio et al. report findings from a community-based hepatitis B virus (HBV) screening program for sub-Saharan African migrants in Catalonia, Spain, utilizing simplified testing and expedited referral to specialist care. Their findings support the adoption of these strategies to increase HBV testing and linkage to care among at-risk populations.

## Introduction

Hepatitis B virus (HBV) is a major public health challenge, resulting in an estimated 820,000 yearly deaths, and is responsible for more than half of all new cases of hepatocellular carcinoma globally^1^. The majority of the burden of HBV can be found in South East Asia and sub-Saharan Africa (sSA), of which HBV in West Africa is the second leading cause of cancer in the region^2^. In 2020, 281 million people were estimated to have migrated to a country besides their own, up 3.5% in comparison to 2019^3^. Migration to the European Union (EU) has increased over the years^4^ and the number of Africans living outside of Africa grew from 17 million in 2015 to 19.5 million in 2020, with migration to Europe being the most pronounced^2^. Spain received around 200,000 migrants in 2020, of which a large proportion were people migrating from countries with an intermediate or high prevalence of HBV, such as countries in sSA and Eastern Europe^5^.

West African migrants make up a large proportion of the migrant population in Catalonia. Late presentation to viral hepatitis specialist care was reported to be 25% in Spain and 55% of the HBV cases were among foreign-born individuals, with 35% presenting late to care^6^, highlighting the need for early diagnosis in this population at risk of developing advanced liver disease. The increase in migration and the resulting relocation of people to new countries comes with a set of unique challenges. Host countries with large numbers of migrants must be prepared to address the health needs of these incoming populations, particularly when migrants are disproportionately affected by specific conditions, including infectious diseases and chronic diseases like viral hepatitis. Migrants have lower rates of vaccination against vaccine-preventable diseases^7^, including against HBV, in comparison to their host populations, and few EU countries have vaccination programs that specifically target older migrants who might have missed their childhood immunizations^8^.

Migrants face structural and sociocultural barriers that may hinder their ability to access the health system, resulting in underutilization or loss of follow-up in their host country’s health system. Despite having the right to access the health system in Catalonia, Spain, migrants, particularly undocumented migrants, make less use of health services in comparison to those with a regular migratory status^9^. Strengthening community-based health programs is a valuable strategy to reduce health inequities and increase linkage to care among this difficult-to-reach population. Implementation of novel testing approaches, including dried blood spot (DBS) testing, in community settings was associated with increased coverage in several settings and among targeted population groups at higher risk for infection, vulnerable and hard-to-reach populations, including migrants^10^. In line with the World Health Organization’s goal to eliminate viral hepatitis as a major public health threat by the year 2030 through an increase in screening and diagnosis by 90%^11^, this study uses point-of-care (PoC) diagnostics and simplified blood sample collection tools in community and faith-based settings to simplify the diagnostic process and increase HBV screening, vaccination, and linkage to specialist care among west African migrants living in Catalonia, Spain.

## Methods

### Study design

**Fig. 1:**
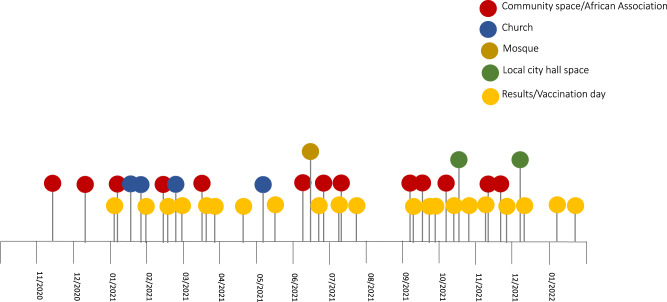
Overview of the HBV-COMSAVA study period and intervention setting for screening. Time of implementation (month/year) and location type. Community space/African association includes leisure spaces, community events, and association offices.

This is a prospective cohort study of West African migrants participating in a community-based HBV screening and vaccination program (HBV-COMSAVA) and preliminary results of the pilot have been published^12^. This intervention used PoC diagnostics in community and faith-based settings in Catalonia, particularly the greater Barcelona area, Spain, to identify migrants living with HBV and link them to specialist care or offer HBV vaccination between 20 November 2020 and 21 January 2022 (Fig. 1). The study team consisted of a coordinator, study nurses for blood sample collection, community health agents (CHAs) who were members of the west African community, and volunteer study surveyors trained in viral hepatitis.

### Study population

Convenience sampling was used and participants were recruited from community study sites, such as churches, mosques, and community spaces provided by community champions and African migrant associations. Community champions are people who have been identified as well-respected members of the community (i.e., imams and preachers) and support the promotion of the project. Inclusion criteria were being 18 years or older, being in possession of a regional public health system card (CatSalut card with an active CIP number), and understanding one of the languages spoken by the study team (Spanish, Catalan, English), including the native languages spoken by the CHAs who are members of the Ghanaian and Senegalese community and speak Twi, Wolof, and French, respectively. Given that the main objective of this study was to screen, diagnose, and link to care (for new cases of HBV), exclusion criteria included having an HBV diagnosis and reporting currently being followed up in regular care and/or being treated for HBV.

### Sample size calculation

Excluding people migrating from Morocco, which make up the largest proportion of African migrants in Barcelona, there were 81,645 persons of African origin reported to be living in Catalonia in 2021, of which 7,056 were Ghanaians and 22,519 were Senegalese^13^. In order to have a sample size of sSA migrants with 95% confidence and ±5 points, a sample size of 383 was needed^14^.

### Clinical samples, data collection, and patient referrals

**Fig. 2:**
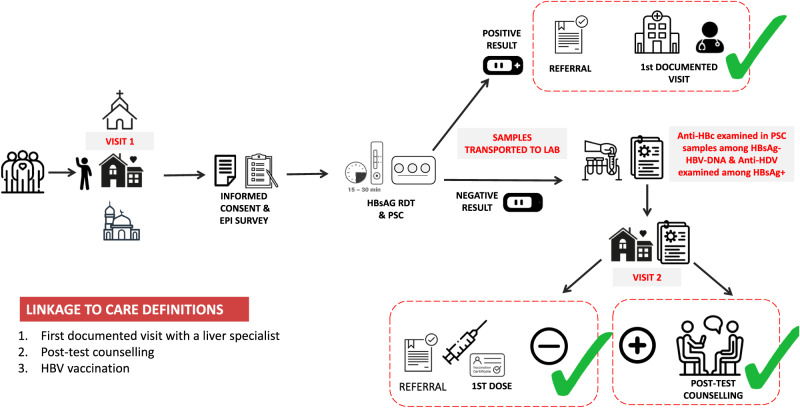
The HBV-COMSAVA care pathway. Hepatitis B surface antigen (HBsAg); plasma separation card (PSC); Hepatitis Delta antibodies (anti-HDV); antibodies against hepatitis B core antigen (anti-HBc); rapid detection test (RDT).

To participate in the study, participants provided written informed consent after a verbal review of the informed consent form. Informed consent was available in Spanish and English, and the study CHAs were always on site to provide intercultural mediation and translation if needed. Participants' voluntary participation and the possibility of withdrawal from the study were informed verbally in addition to being included in writing in the informed consent form. Participants were reminded at the beginning and throughout the interview that they were not required to answer any personal information they were uncomfortable with and could still undergo testing without needing to answer any questions from the epidemiological questionnaire. The six-section epidemiological questionnaire included questions on (1) sociodemographic variables; (2) migration data; (3) HBV previous testing; (4) HBV risk factors, including possible mother-to-child transmission; (5) vaccination status; and (6) presence of other conditions, including HIV and sexually transmitted infections. A list of all variables used and their codification can be found in Supplementary Methods 1. A blood sample was collected intravenously, and approximately 140 μL was spotted from the syringe onto each of three spots (420 μL total) of a plasma separation card (cobas® plasma separation card (PSC), Roche Diagnostics) for analysis in the laboratory. Rapid detection test (RDT) results were delivered during the first community visit to all participants. From the same syringe, 50 μL was spotted onto an RDT (Determine HBsAg 2, Abbott Laboratories) to screen for the presence of hepatitis B surface antigen (HBsAg). This RDT meets the WHO International HBsAg Standard for pre-qualification with an analytical sensitivity of 0.1 IU/mL^15^. Among those who had a reactive HBsAg RDT result, the laboratory examined HBV viral load (HBV-DNA) (cobas® HBV test, Roche Diagnostics) and hepatitis D virus antibodies (anti-HDV) (LIAISON® XL MUREX Anti-HDV, DiaSorin) from the PSC sample^16,17^ The lower limit of detection for HBV-DNA on PSC ranges from 200 to 1000 IU/mL (not well established)^18^. On the same day of the screening (visit 1), participants were offered a direct referral to a hepatologist from participating university hospitals during this initial visit, irrespective of laboratory results (Fig. 2). At the hospital, liver specialists performed a complete blood workup, including HDV co-infection evaluation, and explored fibrosis staging at the initial consultation.

Among participants who did not have a reactive HBsAg RDT result, past resolved HBV infection was determined through exploration of HBV core antibodies (anti-HBc) (Elecsys® Anti-HBc II, Roche Diagnostics) from the PSC sample. PSCs were collected and transported at room temperature to Vall d’Hebron University Hospital Laboratory Liver Pathology Unit (Barcelona) for analysis from eluted plasma 2–7 days after sample collection, and the results were communicated to participants during the second scheduled community field visit up to 30 days after the initial community screening visit (visit 2). During the second community visit, participants who had evidence of past-resolved infection were offered post-test counseling and were provided with an informational brochure explaining the meaning of having a past-resolved infection. Those who had no evidence of a past resolved infection and reported being unvaccinated against HBV were offered the first dose of the HBV vaccine in situ during this second community visit and were provided a referral for the subsequent two doses at their primary care centers.

All patient-reported data collected from the epidemiological survey, human-read RDT results, and laboratory reports from participant blood samples collected with the PSC were recorded in a server-based Microsoft Excel database using pseudoanonymous study ID codes. Double entry was used to ensure the accuracy of the imputed values.

### Outcome: linkage to care

Successful linkage to care was defined as having a first documented visit with a hepatologist in tertiary care for those with an active HBV infection, receiving post-test counseling during the second community visit after laboratory testing for those with an identified past resolved infection, or accepting the first dose of the HBV vaccine in situ during the second community visit.

The number of days from the initial HBV screening in the community to linkage to care was reported as a continuous variable. Linkage to care among those requiring specialist care and those requiring post-test counseling or vaccination were reported separately.

### Statistical procedures

Data were entered into Microsoft Excel and then imported to StataCorp statistical software v17.0. Baseline characteristics of participants were described and summarized, including patient age, sex, country of origin, education level, number of children, employment, years living in Spain, and HBV-related risk factors. Means with standard deviation (SD) were reported for continuous variables with normal distribution, and medians with ranges were reported for variables with non-normal distributions. Frequencies and percentages were calculated for categorical variables.

Pearson’s chi-square test or Fisher’s exact test was used to compare proportions. Student’s *t*-test was used to compare mean values for continuous variables with normal distribution or a Mann–Whitney *U* test for those with non-normal distributions. Proportions with 95% confidence intervals were measured to assess the prevalence of HBV. A *P* value of <0.05 was considered statistically significant for all analyses. A logistic regression model was applied to determine the association between HBV status and linkage to care and unadjusted odds ratios (ORs) and their 95% confidence intervals were reported. Box and whisker charts were used to graphically represent the median, 25th, and 75th percentile in a number of days for successful linkage to care in participants being linked to participating tertiary hospitals and receiving results in a second community visit.

### Ethical considerations

This study received ethical clearance in 2020 from the Ethical Committee of the Hospital Clínic de Barcelona, Barcelona, Spain (n. HCB/2020/1036). Participants were recruited and data was collected until linked to care; therefore, the study was approved as an observational study by the institutional review board. This study was performed in accordance with relevant guidelines and regulations. All participants provided informed written consent. Study information sheets and informed consent forms were available in Spanish and English.

### Reporting summary

Further information on research design is available in the Nature Portfolio Reporting Summary linked to this article.

## Results

**Fig. 3:**
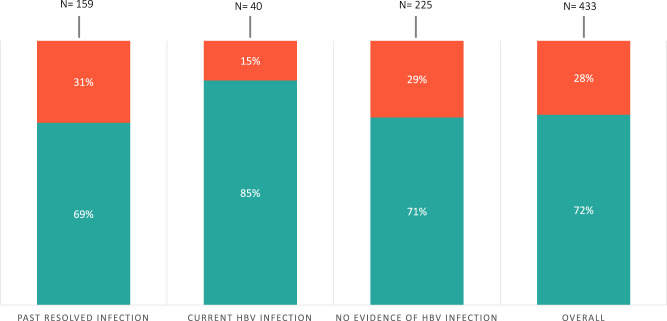
Overall successful linkage to care (blue) among participants and by infection status. Current HBV infection linkage to care was reported among those with a confirmed documented visit in one of the participating tertiary hospitals (*n* = 24/28).

Between November 2020 and January 2022, a total of 444 people were screened during 42 scheduled interventions in community sites, of which 433 were included for analysis (Supplementary Fig. 1). Participants were primarily male (261; 60.3%), from Ghana (315; 72.7%), and with a mean age of 41 years (SD: 10.3). Participants had, on average, 2.3 (SD: 1.6) children. A large majority of the participants had never been (294; 67.9%) or were unsure (60; 13.9%) about being tested for HBV before. The overall HBsAg prevalence was 9.2% (*n* = 40) (95% CI: 6.8–12.4%) and was higher in men compared to women (11.5% vs. 5.8%; *p* = 0.046). Almost half (19; 47.5%) of those HBsAg positive had detectable HBV-DNA levels on PSC, and two (5%) participants were positive for anti-HDV but did not have active HDV infection determined by a full blood work-up carried out once linked to care at a tertiary hospital. Evidence of past resolved infection (HBsAg−/anti-HBc+) was 41.4% (*n* = 159) (95% CI: 36.6–46.4%) among those who were HBsAg-negative (*n* = 393) (Supplementary Data 1). Women were slightly more likely to have evidence of past resolved infection in comparison to men (45.2% vs 38.8%; *p* = 0.207). Overall, 72.3% of participants were successfully linked to care (Fig. 3).

### Linkage to care

**Fig. 4:**
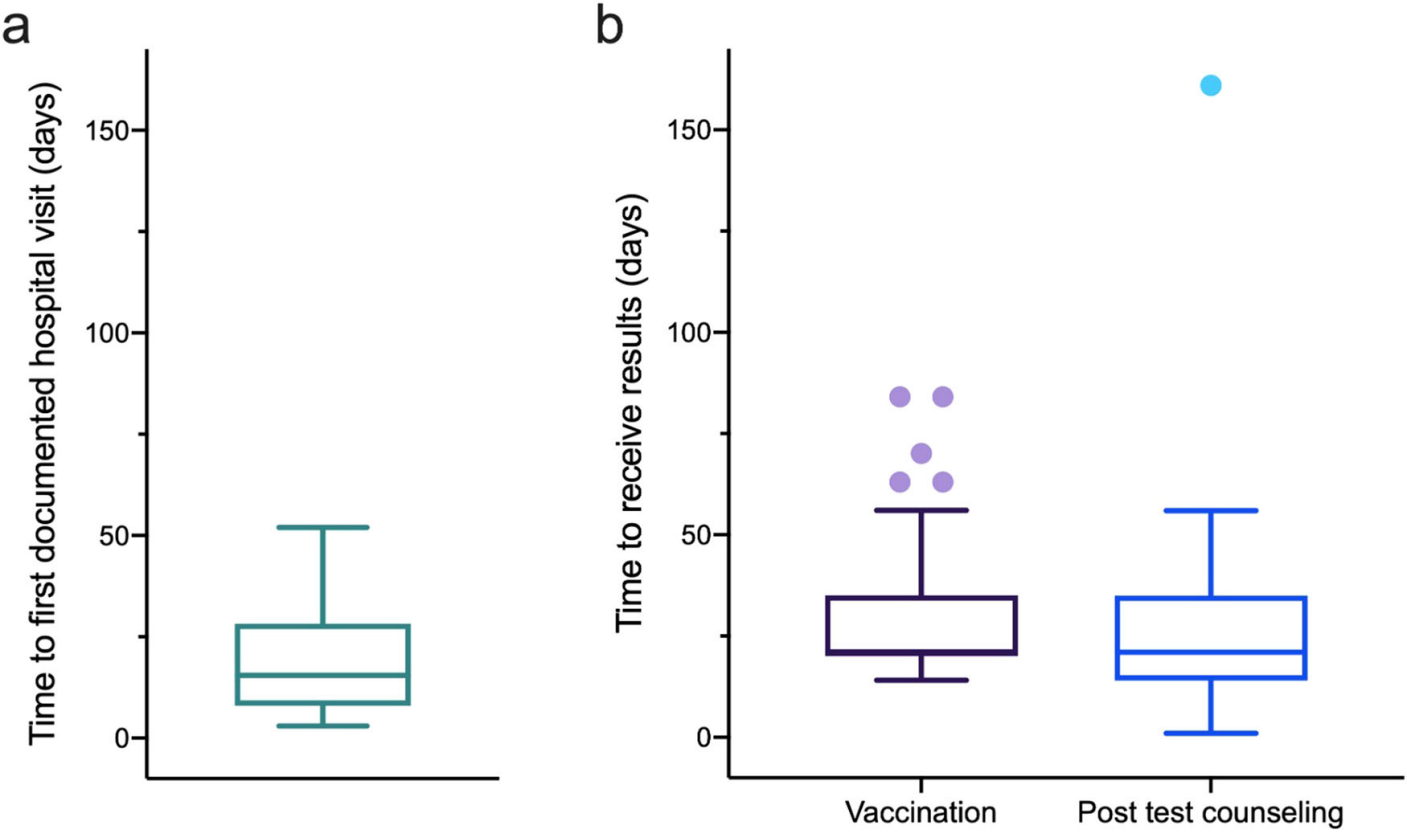
Median number of days for successful linkage to care among participants. Box and whisker chart indicating the median, 25th, and 75th percentile in number of days for successful linkage to care in participants being linked to participating tertiary hospitals (**a**) and those returning for a second visit (**b**) in the community for vaccination or post-test counseling.

Among those who required a referral to specialist care (*n* = 40), 77% (*n* = 31) had a confirmed first documented visit with a specialist as of the end of the study period. Of those that were referred, 70% (*n* = 28) were referred to a tertiary hospital affiliated with the study through the direct referral program and among these, 85% (*n* = 24) had a first documented visit with a hepatologist. Clinical baseline characteristics of participants who attended a first visit with a participating tertiary hospital and received a full analytical workup are presented in Table 1. Among these participants, the median number of days it took for participants to have a first visit hospital was 15.5 days (IQR: 8–27.5) (Fig. 4). Those requiring specialist care had 5.2 greater odds of being successfully linked to care in comparison to those requiring vaccination or post-test counseling in the community (OR: 5.2, 95% CI: 1.6–17.2).

**Table 1:** Clinical baseline characteristics of participants who attended a first visit with a participating tertiary hospital (*n* = 22), 2020–2022.

Number	Age	HBV-DNA (IU/mL)	Anti-HBe^a^	HBsAgQ	Anti-HDV	HDV-RNA	ALT^b^	AST^b^	Fibrosis stage
1	48	1340	POS	25450	NEG	N/A	53	40	F0–F1
2	53	2050	POS	5932	NEG	N/A	33	34	F0–F1
3	44	280	POS	14537	NEG	N/A	40	39	F0–F1
4	35	723	POS	6002	NEG	N/A	15	19	F0–F1
5	33	14200	POS	12730	NEG	N/A	30	27	F0–F1
6	30	1820	POS	8037	NEG	N/A	59	39	F0–F1
7	34	<20	POS	760	NEG	N/A	44	33	F0–F1
8	47	304	POS	2865	NEG	N/A	36	30	F0–F1
9	52	5440	POS	2416	NEG	N/A	13	18	F0–F1
10	38	1560	POS	6002	NEG	N/A	25	23	F2
11	35	<20	POS	728	NEG	N/A	31	35	F0–F1
12	43	174000	POS	2946	POS	NEG	28	27	F1
13	35	1930000	POS	13069	POS	NEG	178	85	F2
14	31	759	POS	17809	NEG	N/A	23	29	F0–F1
15	48	50	POS	1143	NEG	N/A	64	38	F0-F1
16	30	188	POS	NP	NEG	N/A	13	24	NP
17	34	200	POS	10621	NEG	N/A	14	24	F0–F1
18	33	944	POS	NP	NEG	N/A	40	36	F0–F1
19	33	88	POS	NP	NEG	N/A	20	12	F0–F1
20	33	376	POS	5262	NEG	N/A	18	28	F0–F1
21	45	NP	NP	NP	NP	NP	NP	NP	F0–F1
22	24	238	POS	5	NEG	N/A	21	23	NP

*Note*: Antibodies against Hepatitis B e Antigen (anti-HBeAg); quantitative HBsAg value (HBsAgQ); hepatitis delta antibodies (anti-HDV); hepatitis delta RNA (HDV-RNA); aspartate transaminase (AST); alanine transaminase (ALT); negative (NEG); positive (POS); not performed (NP); not applicable (N/A). (a) All subjects were HBeAg-negative; (b) the upper limit of normality (ULN) for ALT and AST values was set at 40 IU/mL. Of the 24 participants with a documented first visit with a collaborating tertiary hospital, one person left Spain and did not receive a full blood workup and is not reported here; another person was referred to a hospital closer to their home, and ID number 21 did not receive a full blood workup done but was evaluated for fibrosis.

Of those who required HBV vaccination (*n* = 204), over 70% (*n* = 145) received their laboratory test results during the second community visit and 85% (*n* = 123) accepted the first dose of the HBV vaccination series in situ. Post-test counseling informing of past resolved infection or of continued prevention methods (due to participants reporting past HBV vaccination) was required for 174 participants. The majority of participants who received post-test counseling needed to be informed of a past-resolved infection (*n* = 159), and of the 45 people who reported being fully (3 doses) previously vaccinated against HBV, 15 of them were also anti-HBc positive. Of those that needed post-test counseling, 70% (*n* = 130) received it. The median number of days from the first community intervention day to reception of post-test counseling for those requiring vaccination was 21 days (IQR: 14–35), with no differences in median time between the two groups observed (Fig. 4).

Women were less likely to be successfully linked to care in comparison to men (65.1% vs 77.0%; *p* = 0.007), overall. This trend held true for women needing vaccination (62.8% vs 76.3%; *p* = 0.030), linkage to specialist care (80% vs 96%; *p* = 0.083), and post-test counseling (66.2% vs 71.6%; *p* = 0.464) although the latter two were not statistically significant.

### Migration

Recently arrived migrants did not have a significant difference in the proportion of past or current HBV infection in comparison to those who had been in living in Spain for five years or more. However, migrants who had been living in Spain for 11–16 years had the highest proportion of HBsAg-positivity in comparison to other categories of years spent in Spain (15.4%; *p* = 0.038) (Table 2).

**Table 2:** Number of years living in Spain and proportion of past or current HBV infection.

	Total	Current infection			Past or current infection		
	*N* = 433	*N*	%	*P*-value	*N*	%	*P*-value
0–5 years	106	7	6.6	**0.038**	45	43.3	0.438
6–10 years	52	4	7.7		20	39.2	
11–16 years	136	21	15.4		66	49.3	
17+ years	139	8	5.8		68	50.4	

*Note*: Bolded *p*-value signifies statistical significance *p* < 0.05.

Years living in Spain did not show significant differences in successful linkage to care (*p* = 0.417); however, those living in Spain for less than 5 years tended to be less frequently linked to care (66.9% vs 75%) in comparison to those living in Spain between 6 and 10 years, 71.3% were successfully linked to care among those living in Spain between 11 and 16 years, and 76.3% for those living in Spain for ≥17 years).

## Discussion

Migrants from sSA in Spain have a higher risk of living with HBV. This population group engages in the health system less frequently, despite having universal access to health care in the autonomous community of Catalonia, and may, therefore, be unaware of their HBV status. To our knowledge, this is the first study in Spain to offer community-based hepatitis B screening and vaccination for sSA migrant populations. This pilot program showed that offering decentralized testing and vaccination and linkage to care through an expedited referral process to tertiary care for this at-risk group was feasible, with high overall linkage to care rates for those HBsAg positive and requiring specialist care (85%). Results of this study can provide further evidence of the importance of tailored models of care to increase HBV diagnosis, which is currently 10% globally^19^. Further, while this study focused on HBV screening and linkage to care, the infrastructure in place can contribute to increasing integrated community-based care and be scaled up to include other comorbidities in the future.

The complexity of the current model of hepatitis care in Catalonia presents barriers to migrant groups disproportionately affected by viral hepatitis. Community-based testing services for HBV offer adaptable and approachable services for people who may not have frequent contact with the formal health system or who may oftentimes face structural, cultural, or linguistic barriers to accessing care, including primary and tertiary care^20^. In 2022, the Catalan health system reported the number of days to see a specialist after receiving a referral to be as high as 172 days for urology and 111 days for other specialties^21^. The average amount of days to see a liver specialist participating in this model of care was five times less (15.5 days) than within the public health system’s regular pathway. The benefit of community-based testing for high-risk populations has been well described in the viral hepatitis literature, and other programs in Spain^22^ and Europe^23^ follow this model to increase testing uptake. These studies, however, do not offer decentralization of HBV vaccination and refer participants to facility-based care for the entire vaccination scheme, which can result in substantial drop-off along the care cascade. Our study showed that more than 70% of those who required HBV vaccination returned for their results, and 85% of those who presented to the second community visit accepted the first dose in the HBV vaccination series. HBV vaccination is recommended in guidelines for HBV testing^24^ (but not for management)^25^, guidelines for vaccine-preventable diseases^26^, and in the WHO Viral Hepatitis Global Health Sector Strategy (GHSS)^12,27^, which sets increasing HBV vaccination as a target for eliminating viral hepatitis as a major public health threat. Certain migrant populations have been under-immunized for vaccine-preventable infections, and strategies to improve engagement with these groups is crucial to meet the WHO elimination targets and for countries to meet their vaccination targets^28^.

The high linkage to care rates in this study can be attributable to, in part, the use of simplified diagnostic tools, including a rapid lateral flow test and plasma separation cards^18,29^ for blood sample collection as a means to simplify the HBV diagnostic process. Blood samples were obtained through phlebotomy by study nurses and were appropriate for the community setting. To ensure linkage to care, participants who had a reactive rapid test (HBsAg + ) were referred directly to a liver specialist, irrespective of their HBV–DNA levels. This direct referral process yielded a first documented visit in 85% of those who were positive, and 70% of these were with one of the study’s collaborating hospitals that agreed to receive patients on a specific day every week without prior appointments.

In Spain, many hospitals do not accept referrals that have not previously gone through primary care. This restriction could be lifted in order to facilitate streamlined linkage to care, particularly for vulnerable populations. While initial visits were largely successful, continued follow-up of these people has proven difficult in some instances^30^. In one setting in our study, all participants reached the first visit with the liver specialist; however, they did not complete the following scheduled visits for management follow-up. In the United States, a domicile HBV follow-up program for refugees also showed that linkage and retention to care only reached 21% across three US cities^31^. Chronic HBV requires long-term management, which can be difficult to continue among highly mobile migrants or those who may experience barriers to maintaining engagement in care. One study in the Netherlands described the challenge of successfully maintaining migrants in care even after initial linkage to tertiary care due to language barriers^32^. Similarly, onward mobility to other regions in Spain or in Europe due to work availability might have contributed to the loss to follow-up for some of the participants in the present study.

While recognizing the success of this study, we note some limitations that are important to consider for future scaling-up or adaptation. While the project aims to reduce the number of visits participants need to make to receive their results, having to return for communication of results can result in a loss to follow-up. While nearly three out of four participants were successfully linked to care, strategies to reduce this drop-off should be taken into consideration, including PoC tests for biomarkers like anti-HBc or anti-HBs^33^. If these PoC tests become available and utilized in community settings, this could remove an additional step in the pathway for participants and vaccination could even be offered on the same day as testing. The PSC is a novel tool to simplify the sample collection process as fingerstick blood may be used, and HBV markers can be determined from these samples with high sensitivity and specificity^16–18^ However, the lower limit of detection for HBV DNA on PSC ranges from 200 to 1000 IU/mL (not well established)^18^, which would not allow this tool to detect patients with low viral loads, but who are also not currently eligible for treatment based on international guidelines. The use of the PSC for HBV–DNA detection would, therefore, be recommended in real-world settings when other monitoring tools are not available.

Regarding anti-HBc detection from PSC, sensitivity was reported at 50% without the presence of HBsAg positivity^18^. As a result, we could have underestimated the true prevalence of past resolved infections and offered an unnecessarily high dose of the HBV vaccine, given that we rely on self-reported vaccination status. However, participants who receive one dose of the HBV vaccine are at no risk of side effects, and when they present for subsequent doses at their primary care center, they will be informed of possible past laboratory reports or vaccination and will not continue onto additional doses. Participants will, therefore, have received a “booster” dose and infer additional immunological protection, which is relevant for this high-risk population. Likewise, this same concept is applicable to those who may report not being vaccinated against HBV but had actually been correctly immunized in the past. Lastly, while the results of this study are relevant for public health interventions among sSA migrants in Catalonia, results may not be representative across all sSA migrant groups or in other regions of Spain or Europe. Programs to increase HBV testing, vaccination, and linkage to care should be tailored to the epidemiological context of every target population.

## Conclusions

This community-based hepatitis B screening program provides evidence of a successful model for identifying and providing screening and care to West African migrant populations at high risk of HBV infection who may otherwise not engage in care. The model of care was effective in ensuring that sub-Saharan African migrants in the greater Barcelona area with active HBV infection were linked to specialist care.

### Supplementary information

Supplemental information

Description of additional supplementary files

Supplementary Data 1

Supplementary Data 2

Reporting summary

## Data Availability

The data that support the findings of this study, including data for the figures, are not openly available due to reasons of sensitivity. The data are available from the corresponding author upon request for fair use. Data are located in controlled access data storage at ISGlobal. Source data for Figs. 3 and 4 are available as Supplementary Data 2.
