# Supplementary material for: Community-based screening enhances hepatitis B virus linkage to care among West African migrants in Spain
Source: Commun Med (Lond). 2023 Dec 14;3:182. doi: 10.1038/s43856-023-00420-8 (PMC10721926; doi:10.1038/s43856-023-00420-8)
Supplement: Supplementary file 1 — Supplemental information [file 43856_2023_420_MOESM1_ESM.docx]

**SUPPLEMENTAL MATERIAL**

**Supplementary Methods 1.** Complete list of study variables and categorization

*Demographics*

Sex was reported as female or male. Age was calculated by the reported year of birth and recategorized into four groups: 18-29 years, 30-39 years, 40-49 years, and ≥50 years. Country of origin was reported by country name and recategorized for analysis into Ghana, Senegal, Other (other African countries). Number of children was reported as a continuous variable and recategorized for analysis into not having children (0), 1-3 children, and >4 children.

*Socioeconomic status*

The education level of participants was recorded as the highest level of education they had completed: no education, primary school completed, secondary school completed, university bachelor’s degree, vocational/trade school, or university master’s degree or higher. For employment, participants were asked to choose one of the following categories: full-time work (40 hours per week), part-time work (less than 40 hours per week), recently unemployed (less than 3 months), unemployed (3 to 12 months), unemployed (more than 12 months), autonomous worker, student, or other. For analysis, employment was recategorized as unemployed (non-student), employed, or student/other.

*Migration-related factors*

The number of years living in Spain was calculated by participants’ reported year of arrival to Spain. For analysis, number of years in Spain was categorized as living in Spain for 5 years or less (considered a recently arrived migrant), 6-10 years, 11- 16 years, and 17+ years.

*HBV-related risk factors*

Recent travel to Africa was recorded as yes or no based on a participant’s response to having travelled to the African continent in the previous six months or planning to travel in the next 12 months.

Ever being incarcerated, having tattoos or traditional scarring, and ever having received a surgical procedure outside of Spain was recorded dichotomously.

*HBV knowledge, vaccination, and testing*

HBV vaccination status and previous HBV testing were based on participants’ answers to ever having been vaccinated for HBV or ever having been tested for HBV, respectively, and were also recorded as yes, no, or unsure. HBV vaccination status also had an “incomplete” option for people reporting incomplete (1 or 2 doses) vaccination. Participants were asked whether they had ever heard of HBV prior to this study and were collected as yes, no, or unsure.

**Supplementary Figure 1.** Inclusion criteria flow diagram


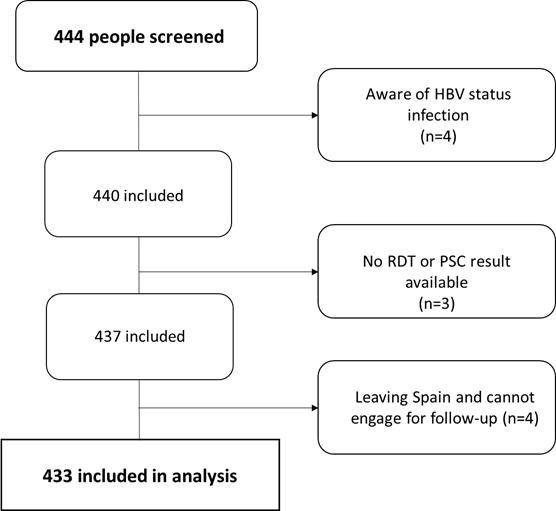


The three people without an RDT or PSC result included two women who it was not possible to collect a venous blood draw due to difficulties in finding their veins and one person whose RDT result did not work and did not want to repeat.
