# Supplementary material for: Community-based screening enhances hepatitis B virus linkage to care among West African migrants in Spain
Source: Commun Med (Lond). 2023 Dec 14;3:182. doi: 10.1038/s43856-023-00420-8 (PMC10721926; doi:10.1038/s43856-023-00420-8)
Supplement: Supplementary file 2 — Description of additional supplementary files [file 43856_2023_420_MOESM2_ESM.docx]

Description of Additional Supplementary Files

**File name:** Supplementary Data 1

**Description:** Participant Characteristics

**File name**: Supplementary Data 2

**Description:** The source data behind the graphs in the paper
